# Exosomal miR‐155‐5p promote the occurrence of carotid atherosclerosis

**DOI:** 10.1111/jcmm.70187

**Published:** 2024-11-04

**Authors:** Wen‐Wen Yang, Qing‐Xiang Li, Fei Wang, Xin‐Ran Zhang, Xian‐Li Zhang, Meng Wang, Dong Xue, Ying Zhao, Lu Tang

**Affiliations:** ^1^ Department of Stomatology, Xuanwu Hospital Capital Medical University Beijing China; ^2^ Department of Oral and Maxillofacial Surgery Peking University School and Hospital of Stomatology Beijing China; ^3^ Department of Vascular Surgery, Xuanwu Hospital Capital Medical University Beijing China

**Keywords:** carotid atherosclerosis, chronic periodontitis, exosome, LPS, miR‐155‐5p

## Abstract

Periodontitis is a significant independent risk factor for atherosclerosis. Yet, the exact mechanism of action is still not fully understood. In this study, we investigated the effect of exosomes‐miR‐155‐5p derived from periodontal endothelial cells on atherosclerosis in vitro and in vivo. Higher expression of miR‐155‐5p was detected in the plasma exosomes of patients with chronic periodontitis (CP) and carotid atherosclerosis (CAS) compared to patients with CP. Also, the expression level of miR‐155‐5p was associated with the severity of CP. miR‐155‐5p‐enriched exosomes from HUVECs increased the angiogenesis and permeability of HAECs and promoted the expression of angiogenesis, permeability, and inflammation genes. Along with the overexpression or inhibition of miR‐155‐5p, the biological effect of HUVECs‐derived exosomes on HAECs changed correspondingly. In ApoE−/− mouse models, miR‐155‐5p‐enriched exosomes promoted the occurrence of carotid atherosclerosis by increasing permeable and angiogenic activity. Collectively, these findings highlight a molecular mechanism of periodontitis in CAS, uncovering exosomal miR‐155‐5p derived periodontitis affecting carotid endothelial cells in an ‘exosomecrine’ manner. Exosomal miR‐155‐5p may be used as a biomarker and target for clinical intervention to control this intractable disease in future, and the graphic abstract was shown in Figure S1.

## 1 INTRODUCTION

Carotid atherosclerosis is the primary cause of stroke and a leading cause of mortality worldwide.^1^, ^2^ Periodontitis is a significant independent risk factor for atherosclerosis^3^, ^4^, ^5^; however, the mechanism through which periodontitis promotes the formation of atherosclerosis is not fully understood. Over the years, several hypotheses have been proposed: (1) periodontal pathogens, especially *Porphyromonas gingivalis* (*P. gingivalis*), translocate into the circulatory system and cause inflammation and plaque formation^2^; (2) lipopolysaccharide (LPS) from periodontal pockets enter the peripheral blood and generate low‐grade endotoxemia.^6^ Preclinical studies have confirmed that exogenous *P. gingivalis* or LPS could induce vascular endothelial cell dysfunction and atherosclerosis in a mouse model.^7^, ^8^, ^9^, ^10^ Yet, the dose of LPS used in mouse experiments was 25–50 μg per mouse,^7^, ^9^ which was much higher than the LPS level (about 0.5–1.5 ng/mL) tested in patients with periodontitis.^11^, ^12^, ^13^ Meanwhile, free LPS cannot passively diffuse to cellular membranes.^14^ The way LPS from periodontal tissues affects distant carotid sites is not fully understood.

Vascular endothelial cells are critical for maintaining vascular homeostasis, and dysfunctional endothelial cells have an important role in the formation of atherosclerosis.^15^ Several studies indicated that LPS could cause periodontal microvascular endothelial cell damage.^16^, ^17^ Also, studies have found that LPS stimulation can induce endothelial cells to release exosomes,^18^, ^19^ which are cell‐derived nanovesicles (40–100 nm in size), containing lipids, RNAs (especially microRNAs) and proteins.^20^ Exosomes are crucial mediators in intercellular communications by transmitting abundant bioactive molecules to recipient cells.^21^ Exosomes have also been demonstrated to play distant roles, affecting distant vascular endothelial cells and changing their functional status.^22^ However, whether exosomes derived from periodontal cells could affect distant carotid endothelial cells and whether this process mediates periodontitis‐induced atherosclerosis remains unclear.

MicroRNAs (miRNAs) are small non‐coding RNAs that regulate gene translation through silencing or degradation of target mRNAs. miRNAs have been associated with many physiological and pathological processes.^23^ For example, miR‐155 is encoded by the MIR155 host gene.^24^, ^25^ Target genes that are regulated by miR‐155 encode immunomodulatory proteins, tumour‐suppressor proteins and inflammatory‐related proteins.^26^ Therefore, miR‐155 is often associated with cardiovascular diseases, inflammation and cancer.^27^, ^28^ A recent study reported that the expression level of miR‐155‐5p in endothelial cells was significantly increased when stimulated by pro‐inflammatory cytokines.^29^ Furthermore, miR‐155‐5p had higher expression in atherosclerotic femoral arteries than normal ones,^30^ which suggests that miR‐155‐5p might have a critical role in atherosclerosis occurrence. However, the specific roles of miR‐155‐5p between periodontitis and CAS remain to be clarified.

In this research, we examined the effect of exosomes‐miR‐155‐5p derived from periodontal endothelial cells on atherosclerosis in vitro and in vivo. Our data found miR‐155‐5p was highly expressed in the plasma exosomes from carotid atherosclerosis (CAS) patients co‐suffered with chronic periodontitis (CP) and in CAS tissue samples. In addition, we discovered that LPS promoted the expression of exosomal miR‐155‐5p, and exosomal miR‐155‐5p derived from periodontal endothelial cells could be transmitted to distant vascular endothelial cells, thus inducing endothelial permeability and angiogenesis. We investigated a novel access of miR‐155‐5p‐enriched exosomes from periodontitis to CAS, thus promoting CAS formation.

## 2 SUBJECTS, MATERIALS AND METHODS

### 2.1 Human tissue samples

Patients diagnosed with chronic periodontitis (CP) or (and) carotid atherosclerosis (CAS) requiring carotid endarterectomy at the Xuanwu Hospital of Capital Medical University in 2022 were enrolled in the current study. The diagnosis of periodontitis was confirmed according to the 2017 world workshop on the classification of periodontal disease: interdental CAL (clinical attachment level) detectable at ≥2 nonadjacent teeth or buccal or oral CAL ≥3 mm with pocketing >3 mm detectable at ≥2 teeth.^31^ CAS was diagnosed by vascular ultrasound, and CAS requiring carotid endarterectomy was further confirmed by a vascular surgeon. Exclusion criteria were the following: patients undergoing periodontal treatment within 6 months, patients aged <18 years old, patients with diabetes, pregnant patients and patients who refused to participate in this study. The plasma samples were obtained from patients with CP but without CAS (*n* = 6) and CP with CAS (*n* = 6) for sequencing analysis, and plasma samples from patients with mild/moderate CP (*n* = 6), severe CP (*n* = 6), mild/moderate CP with CAS (*n* = 6) and severe CP with CAS (*n* = 7) for PCR analysis. CAS tissue samples (*n* = 5) and Normal vessel samples (*n* = 5) adjacent to CAS tissue were obtained for PCR analysis. This study was approved by the Ethics Committee of Xuanwu Hospital, Capital Medical University (NO. LYS[2022]075), and informed consent was obtained from all patients.

### 2.2 Experimental animals

The male ApoE^−/−^mice, 5–6 weeks old, were purchased from Beijing Vital River Laboratory Animal Technology Co., Ltd. All the animals were housed in an environment with a temperature of 22 ± 1°C, relative humidity of 50 ± 1%, and a light/dark cycle of 12/12 h. In addition, all animal studies (including the mice euthanasia procedure) were done in compliance with the regulations and guidelines of Xuanwu Hospital, Capital Medical University institutional animal care and conducted according to the AAALAC and the IACUC guidelines (Permit Numbers: XW‐20220217‐1).

### 2.3 Cell lines and transfection

Human umbilical vein endothelial cells (HUVECs) and human aortic endothelial cells (HAECs) were obtained from the PriCells (Wuhan, China). Cells were cultured in an endothelial cell medium (ECM, ScienCell, USA) supplemented with 10% FBS and 1% Penicillin/Streptomycin in a humidified atmosphere containing 5% CO_2_/95% air at 37°C.

The lentiviral vector PHBLV‐h‐miR‐155‐5p‐GFP‐LUC (used to construct the miR‐155‐5p expression vector) was purchased from HanBio (HanBio Biotechnology, China). After screening, HUVECs stably overexpressing miR‐155‐5p (OE cells) and control cells (Vector cells) were established. For miR‐155‐5p interference, miR‐155‐5p inhibitor and negative control (NC) was bought from Guangzhou RiboBio Co., Ltd. (RiboBio Co., Ltd., China).

### 2.4 Sequencing analysis

The next‐generation sequencing analysis (for microRNA detection) (BGI Shenzhen, China) was used. The recommended cut‐off for the *p* value and fold change were 0.05 and 2, respectively. Heatmap analysis was used to identify differentially expressed microRNAs.

### 2.5 In silico analysis

In order to identify genes that may affect CAS formation, we compared the driver genes ranked by Vogelstein et al. with the target gene of miR‐155‐5p.^32^ MirSNP, is a database predicting miRNA‐mRNA binding sites, was used to find miRNA‐related SNPs.^33^ Next, the potential interaction of miR‐155‐5p with target mRNAs was analysed using TargetScan (http://www.targetscan.org/vert_72/) and miRBase (http://www.mirbase.org/index.shtml). Then, we used RNAhybrid (https://bibiserv.cebitec.uni‐bielefeld.de/rnahybird) to predict the miRNA‐mRNA binding structures.

### 2.6 Cell proliferation and migration assays

Cell viability was assessed with CCK‐8 reagents (Dojindo Laboratories, Japan) according to the manufacturer's instructions. Briefly, cells were seeded in 96‐well plates at 3000 cells/well in 100 μL of culture medium and then exposed to gradually increased concentrations of *P. gingivalis* LPS (0.5, 1.0, 2.0 and 4.0 μg/mL LPS) for 24 h, 48 h and 72 h. At each time point, CCK‐8 solution was added to each well and incubated for another 2 h at 37°C. The absorbance at 450 nm was determined using the Varioskan Flash (Thermo Scientific).

For the migration assay, 5 × 10^4^ cells were seeded on the upper chamber of cell inserts (8.0 μm pore membrane, Falcon) in ECM (10% FBS) with or without 0.5 μg/mL LPS. The inserts were then placed into the lower chamber of a 24‐well plate containing ECM with 20% FBS. After 16 h, any cells remaining in the upper chamber of the insert were removed with a sterile cotton swab. The migrating cells on the bottom surface were stained with 1% crystal violet, examined, counted and imaged using Digital Microscope (Olympus).

### 2.7 Tube formation and endothelial permeability assays

For the tube formation assay, 2 × 10^4^ cells were seeded on matrigel matrix‐coated (Gibco, USA) wells and treated with 0.5 μg/mL LPS or 10 μg/mL exosomes. After 12 h, the tube‐like structures in each well were counted, and the results represented the average number of tube‐like structures in triplicate wells.

For the permeability assay, endothelial cells were cultured on transwell inserts (0.4 μm pore membrane, Falcon) and allowed to form confluent monolayers (typically after 24 h). The medium in the upper chamber was then replaced with a complete medium containing 0.12 mg/mL FITC‐labelled dextran (40 kDa) (Sigma‐Aldrich). At each time point after adding 0.5 μg/mL LPS or 10 μg/mL exosomes, 50 μL of a medium in the bottom chamber was harvested, and fluorescence was measured (excitation 490 nm and emission 520 nm).

### 2.8 qRT‐PCR

Total RNA was extracted from cells, exosomes or tissues using TRIzol reagent (Invitrogen) according to the manufacturer's instructions. RNA was then reverse transcribed into cDNA using a reverse transcription kit (Takara). Quantification of mRNA expression was performed using FastStart Universal SYBR Green Master (ROX) reagent (Roche) on an ABI Step One Plus Real‐Time PCR System. GAPDH or U6 was used for normalization, and relative gene expression was calculated using the 2^−ΔΔCt^ method. Sequences of the primers are shown in Table S1.

### 2.9 Exosome isolation, labeling and uptake

Exosomes from cell supernatants were collected using ultracentrifugation and sucrose cushion.^34^, ^35^ Briefly, cell supernatants were subjected to consecutive centrifugation to remove cellular debris and large vesicles. Supernatants were then passed through a Centrifugal Filter (100 K, Millipore) at 5000 ***g*** for 30 min. The retained, concentrated supernatants were subjected to a 30% sucrose/deuterium oxide (D_2_O) cushion. After gradient centrifugation at 100,000 ***g*** for 70 min using an Optima L‐90 K Ultracentrifuge (Beckman Coulter, USA), the exosome‐enriched sucrose/D_2_O was resuspended in PBS. The retained exosomes were stored at −80°C. The protein contents of exosomes were determined using a Bradford Protein Assay Kit (Beyotime Biotechnology). Exosomes were isolated for the patient or mouse plasma samples using the exoRNeasy Serum/Plasma Maxi Kit (Qiagen) according to the manufacturer's instructions.

Purified exosomes were labelled with a red fluorescent cell linker PKH26 or a green fluorescent cell linker PKH67 (Sigma‐Aldrich) according to the manufacturer's protocol. Exosomes and endothelial cells were fluorescently labelled using PKH26 and Actin‐Tracker Green (phalloidin‐FITC, Beyotime Biotechnology), respectively. Cellular nuclei were stained using DAPI (ZSGB‐Bio). Endothelial cells were incubated with labelled exosomes for 24 h, and imaging of exosome uptake was performed using a TCS‐SP8 DIVE confocal laser scanning microscope (Leica).

### 2.10 Transmission electron microscopy (TEM) and nanoparticle tracking analysis

TEM was used to identify the morphology of exosomes. Exosomes were diluted in 100 μL PBS and kept at 4°C before further analysis. After pretreatment, the samples were examined using an HT‐7800 electron microscope (Hitachi). The exosome size distribution and particle concentration were determined by nanoparticle tracking analysis (NTA). Briefly, the exosomes were diluted with PBS (1:20) and measured using a Flow NanoAnalyzer (NanoFCM).

### 2.11 Flow cytometry

Exosomes were stained with CD63 (BD), CD9 (BD) or respective isotype control antibody (BioLegend) for 30 min at room temperature. After being centrifuged at 100,000 ***g*** for 70 min and resuspended in PBS, exosomes were analysed using a flow cytometer (NanoFCM).

### 2.12 Animal experiments

All experiments were performed on ApoE^−/−^ mice older than 6 weeks of age, and the mice were fed a high‐fat diet (21% crude fat, 0.15% cholesterol and 19.5% casein). For the intravenous injection of exosomes into mice, exosomes (50 μg) were injected intravenously into mice (*n* = 6 per group) every 7 days. On day 49, mice received an intravenous injection of PKH67‐labelled exosomes, and the bioluminescence of mice was measured using the IVIS Imaging System (Caliper Life Sciences) to monitor the biodistribution of labelled exosomes. On day 70, the plaques formation, vasodilation and vasoconstriction diameter in the carotid artery and aorta were detected using a Vevo 3100 ultrasound scanner (FUJIFILM VisualSonics). After 12 weeks, the mice were sacrificed, and blood was collected from the retro‐orbital venous plexus into EDTA tubes. For the local injection of LPS into mice, mice in LPS group were injected with 20 μL (10 μL on each side) of *P*. *gingivalis* LPS (1 mg/mL) at the buccal gingival sulcus of bilateral maxillary second molars of mice (once a week for 6 weeks). On day 42, the mice were sacrificed, and blood (*n* = 6 per group) was collected from the retro‐orbital venous plexus into EDTA tubes.

The right carotid artery and abdominal aorta samples were then snap‐frozen in liquid nitrogen for RNA extraction. The excised thoracic aorta samples were washed in PBS, post‐fixed in 4% paraformaldehyde, and then collected for Oil‐Red O staining. The left carotid artery samples were fixed in 4% paraformaldehyde and then embedded in paraffin for H&E analysis.

### 2.13 Analysis of stained sections

Plaque extensions on the tissue of the thoracic aorta were evaluated using en face oil red O staining. Briefly, after the fixed aortas were cut open to expose the atherosclerotic plaques, the tissues were rinsed in water for 10 min and then in 60% isopropanol. The aortas were stained with Oil Red O for 20 min, then rinsed in 60% isopropanol, followed by rinsing three times in water. The images were recorded using a camera, and atherosclerotic areas were quantified by Image J software. Plaques on tissue sections of the aortas were determined by measuring the area of oil red O staining using Image J software. Paraformaldehyde‐fixed, paraffin‐embedded carotid artery tissues were analysed by H&E staining for morphology. The sections were analysed to identify the region with maximal luminal narrowing and selected for morphometric measurement of the medial area, the intimal area and the intima/media (I/M) ratio.

### 2.14 Statistical analysis

All statistical analyses were carried out with SPSS Version 19.0 (IBM). All numerical data were represented by mean ± SD unless otherwise noted. Data were statistically analysed by a 2‐tailed *t*‐test or ANOVA unless otherwise indicated. All numerical data were from at least three independent experiments, and a *p* value <0.05 was considered statistically significant. The flowchart for purpose and related methods was shown in Figure S2.

## 3 RESULTS

### 3.1 MiR‐155‐5p is overexpressed in CAS and CP plasma exosomes and in CAS tissue samples

**FIGURE 1:**
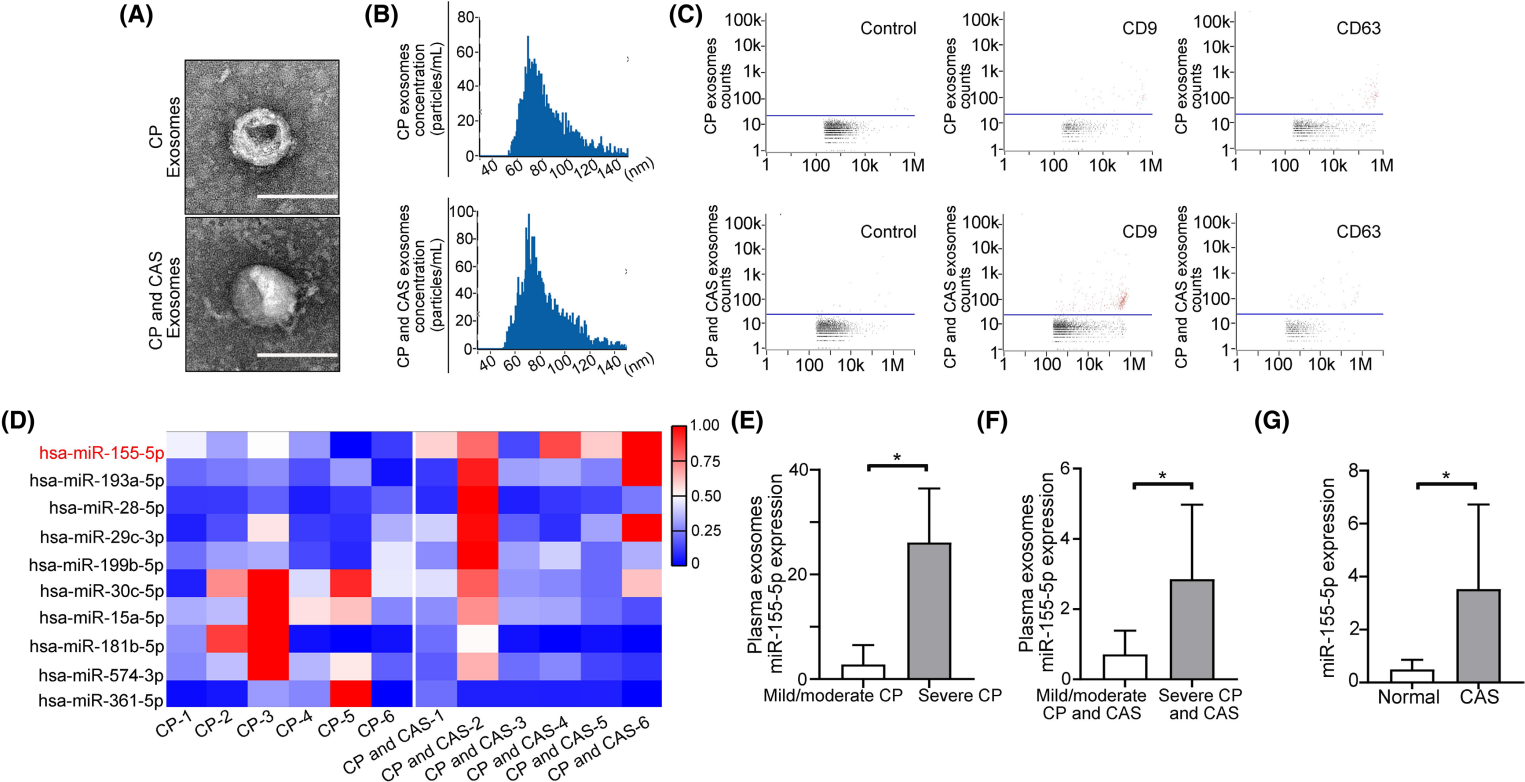
Expression of miR‐155‐5p is upregulated in plasma exosomes from patients with CP and CAS. (A) Electron microscopy images of human plasma exosomes. Scale bar = 100 nm. (B) NTA was used to determine the particle size and concentration of human plasma exosomes. (C) Flow cytometric analysis of CD9 and CD63 (exosomal marker) expression in human plasma exosomes. (D) The heatmap of microRNAs expressed in plasma exosomes from patients with CP or patients with CP and CAS (*n* = 6 per group). (E, F) Plasma exosomes were isolated from patients with mild or moderate CP (*n* = 6), severe CP (*n* = 6), mild or moderate CP and CAS (*n* = 6) and severe CP and CAS (*n* = 7). Real‐time PCR was used to determine the miR‐155‐5p expression levels. (G) Real‐time PCR was used to determine the miR‐155‐5p expression levels in Normal (*n* = 5) and CAS (*n* = 5) tissue samples. All data are expressed as mean ± SD, *p < 0.05.

Under physiological conditions, vascular endothelial cells can release a considerable number of exosomes, which are carried throughout the body by the blood to perform biological functions. To verify whether exosomes from patients with CP were involved in the formation of carotid atherosclerosis (CAS), plasma exosomes from patients only with CP or with both CP and CAS were isolated. An electron microscope and nanoparticle tracking analysis (NTA) were used to determine the patient plasma exosomes' particle size and concentration (Figure 1A,B). The NTA results showed that the plasma exosomes from patients with CP and CAS were present in higher concentrations at the most abundant particle size compared to patients with CP only. The flow cytometric analysis was then used for exosome identification (Figure 1C), and microRNA sequencing was carried out to explore the differences between plasma exosomes from CP patients and CP/CAS patients. According to the sequencing results, miR‐155‐5p was the most significantly differentially expressed microRNA. Also, miR‐155‐5p was higher in the CP + CAS group versus CP group (approximately 3 times, Figure 1D).

Afterward, we isolated plasma exosomes from different degrees of CP patients and CAS patients with different degrees of CP, then we detected the expression level of miR‐155‐5p. Higher miR‐155‐5p levels were observed in plasma exosomes from severe CP patients than mild or moderate CP patients (Figure 1E). Compared with mild or moderate CP and CAS patients, exosomes derived from severe CP and CAS patients contained more miR‐155‐5p (Figure 1F). High miR‐155‐5p mRNA expression levels were observed in CAS tissue samples (Figure 1G). Collectively, miR‐155‐5p was overexpressed in CP and CA plasma exosomes and was associated with the severity of CP.

### 3.2 LPS increases angiogenesis and permeability of endothelial cells

**FIGURE 2:**
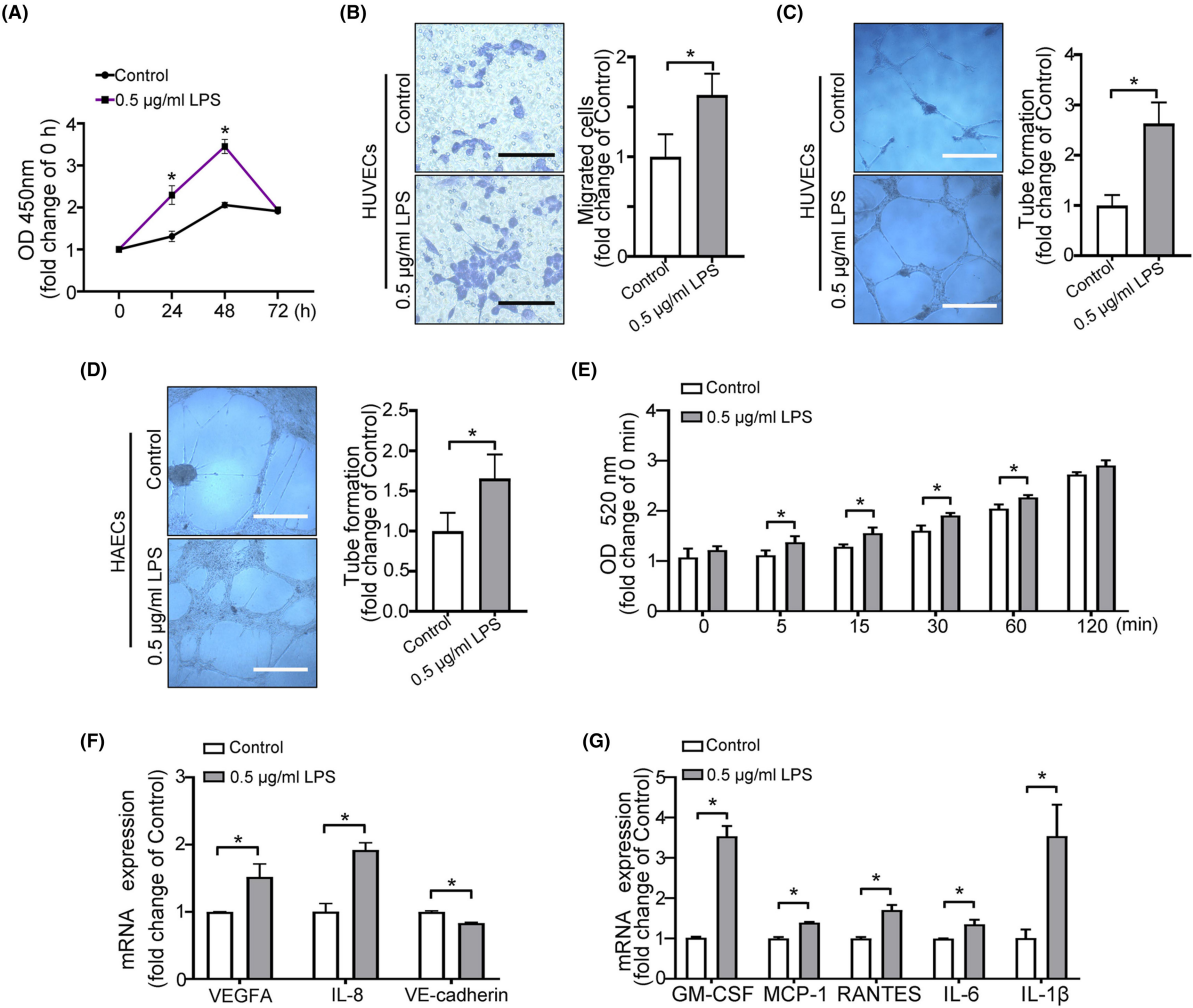
LPS is involved in the angiogenesis and permeability of endothelial cells. (A) CCK‐8 assays were used to assess HUVECs after incubation with or without 0.5 μg/mL LPS. (B) Migration assay for HUVECs after incubation with or without 0.5 μg/mL LPS. Scale bar = 200 μm. (C, D) Tube formation assays for HUVECs and HAECs after incubation with or without 0.5 μg/mL LPS. Scale bar = 500 μm. (E) Permeability assay for HUVECs after incubation with or without 0.5 μg/mL LPS. (F, G) qRT‐PCR analysis of relative VEGFA, IL‐8, VE‐cadherin, GM‐CSF, MCP‐1, RANTES, IL‐6 and IL‐1β mRNA expression levels in HUVECs cultured with 0.5 μg/mL LPS. All data are expressed as mean ± SD, *p < 0.05.

In order to explore the effects of chronic periodontitis on vascular endothelial cells, we performed simulation experiments using HUVECs in vitro. The proliferation ability of HUVECs was significantly increased under 0.5 μg/mL LPS treatment (Figure S3, Figure 2A). Moreover, 0.5 μg/mL LPS stimulation prominently enhanced cell migration, angiogenic capacity and permeability of HUVECs (Figure 2B–E). Under the same treatment conditions, HAECs also acquired stronger angiogenesis ability (Figure 2D).

Subsequently, qRT‐PCR experiments further confirmed these results at the gene level. As shown in Figure 2F, the expression levels of angiogenesis‐related genes (VEGFA and IL‐8) in HUVECs were increased after treatment with LPS. In addition, the expression levels of VE‐cadherin, which have key roles in vascular permeability, were decreased. Furthermore, LPS increased inflammation factors GM‐CSF, MCP‐1, RANTES, IL‐6 and IL‐1β mRNA levels (Figure 2G). These results suggested that LPS could increase the angiogenesis and permeability of endothelial cells.

### 3.3 LPS induces the enrichment of miR‐155‐5p in exosomes

**FIGURE 3:**
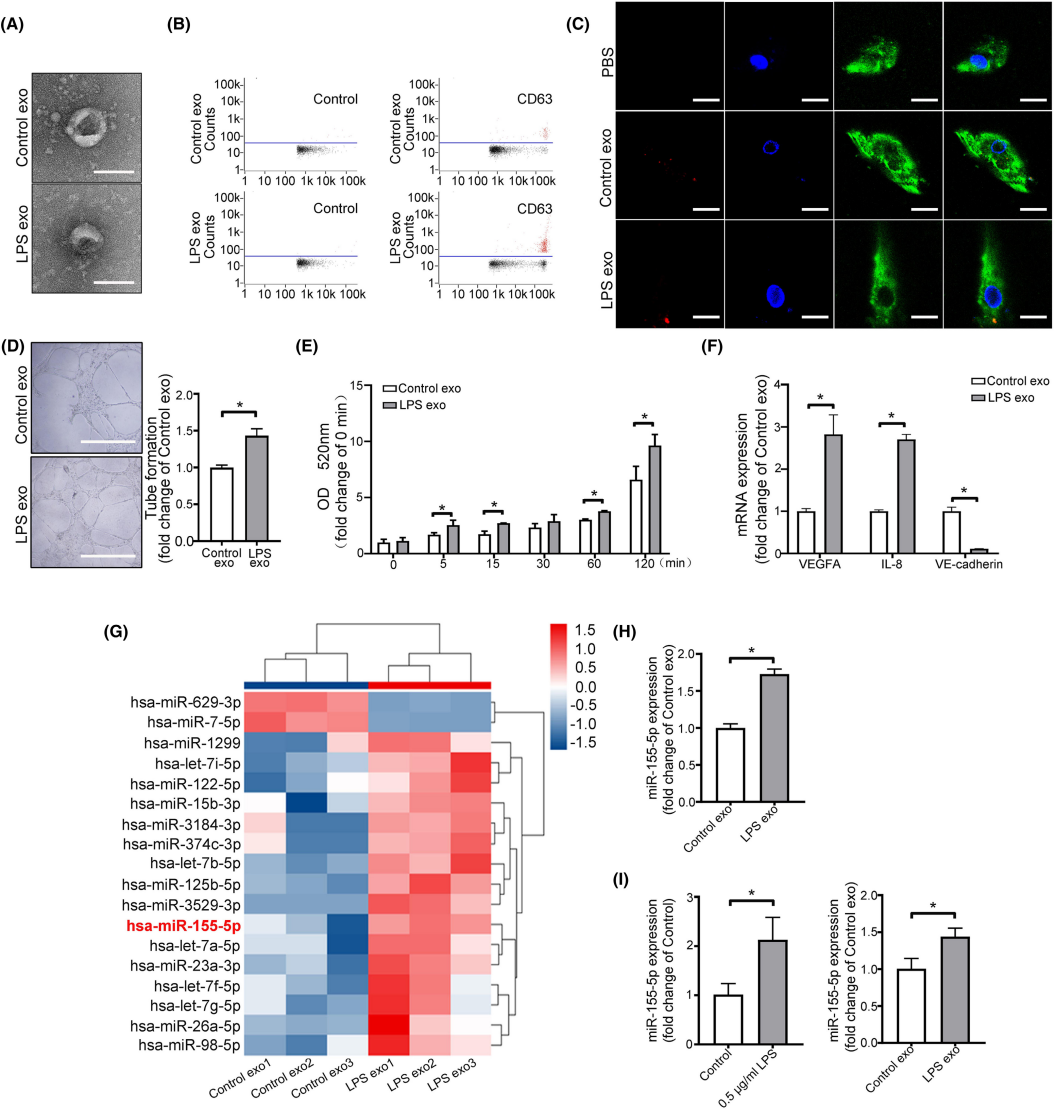
Exosomes derived from HUVECs cultured with 0.5 μg/mL LPS promote angiogenesis and permeability and increase the miR‐155‐5p level of HAECs. (A) Electron microscopy images of exosomes from HUVECs cultured with or without LPS (named LPS exo or Control exo). Scale bar = 100 nm. (B) Flow cytometric analysis of CD63 (exosomal marker) expression in Control exosomes and LPS exosomes. (C) The uptake of PKH26‐labelled exosomes (red) by HAECs was identified using fluorescence confocal microscopy. Scale bar = 10 μm. (D) Images of tube formation of HAECs after incubation with Control exosomes or LPS exosomes. Scale bar = 500 μm. (E) Permeability assay for HAECs after incubation with Control exosomes or LPS exosomes. (F) qRT‐PCR was used to assess the relative VEGFA, IL‐8 and VE‐cadherin expression levels in HAECs after stimulation with Control exosomes or LPS exosomes. (G) The heatmap and hierarchical clustering of microRNAs differentially expressed in Control exosomes or LPS exosomes. Red, high relative expression; blue, low relative expression. (H, I) qRT‐PCR analysis of relative miR‐155‐5p expression levels in Control exosomes and LPS exosomes, and miR‐155‐5p levels in HAECs after incubation with 0.5 μg/mL LPS, Control exosomes, or LPS exosomes. All data are expressed as mean ± SD, *p < 0.05.

Subsequent experiments displayed the effect of LPS on HUVECs‐derived exosomes. With or without 0.5 μg/mL LPS stimulation, HUVECs released exosomes (LPS exo and Control exo) that were smaller than 100 nm (Figure 3A,B). After labeling cells with PKH26, it was found that both Control exosomes and LPS exosomes could be uptaken by HAECs (Figure 3C). Further experiments showed that tube formation ability and permeability of HAECs were obviously increased after treatment with LPS exosomes, compared with the control exosomes (Figure 3D,E). Correspondingly, HAECs expressed more VEGFA, IL‐8 and less VE‐cadherin after treatment with LPS exosomes (Figure 3F).

In order to confirm whether miR‐155‐5p was the key factor in LPS exosomes, microRNA sequencing of Control exosomes and LPS exosomes were analysed. As shown in Figure 3G, LPS induced the enrichment of miR‐155‐5p in exosomes, which was consistent with the results based on clinical samples in Figure 1D. As supporting evidence, we showed that miR‐155‐5p was overexpressed in LPS exosomes (Figure 3H), and treatment with LPS or LPS exosomes increased miR‐155‐5p levels in HAECs (Figure 3I).

### 3.4 MiR‐155‐5p enhances angiogenesis and permeability of endothelial cells

**FIGURE 4:**
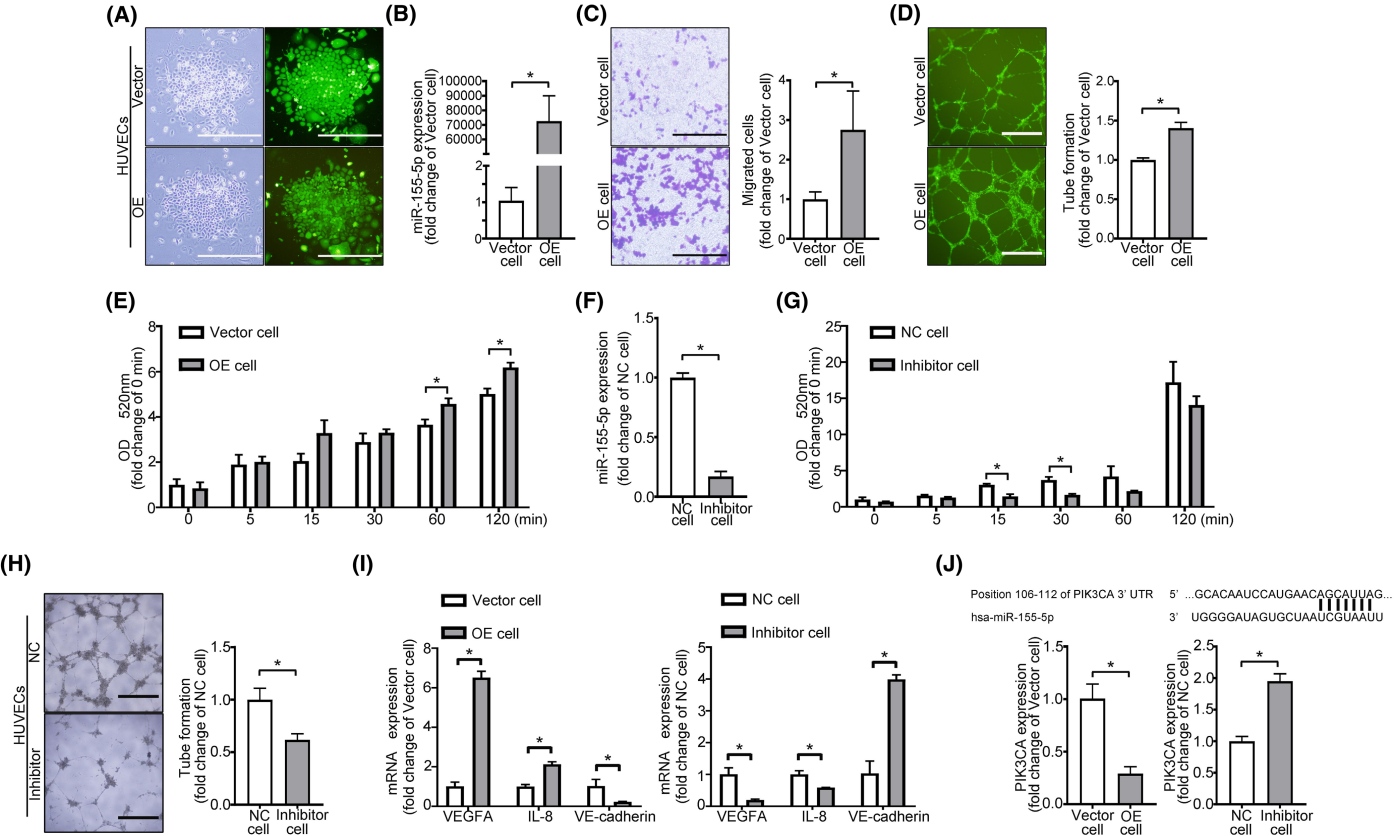
miR‐155‐5p promotes endothelial cells' angiogenesis and permeability. (A) Fluorescence microscopy of HUVECs stably transfected with the control vector (Vector cells) or the miR‐155‐5p expression vector (OE cells). (B) Expression of miR‐155‐5p in Vector or OE cells. (C) Migration assays of Vector and OE cells. (D) Representative images of tube formation of Vector and OE cells. (E) Permeability assays for Vector and OE cells. (F) Expression of miR‐155‐5p in HUVECs transiently transfected with miR‐155‐5p inhibitor (Inhibitor cells) or negative control (NC cells). (G, H) Permeability and tube formation assays of NC and Inhibitor cells. (I) qRT‐PCR analysis of VEGFA, IL‐8, and VE‐cadherin expression levels in Vector, OE, NC or Inhibitor cells. (J) The binding sites of miR‐155‐5p and PIK3CA and the expression of PIK3CA in Vector, OE, NC or Inhibitor cells by qRT‐PCR. All data are expressed as mean ± SD, *p < 0.05.

To investigate the function of miR‐155‐5p, we generated OE cells and Vector cells (Figure 4A,B). Overexpression of miR‐155‐5p in OE cells increased migration, capillary formation and permeability of HUVECs (Figure 4C–E), while the knockdown of miR‐155‐5p (Inhibitor cells) and control cells (NC cells) (Figure 4F) reversed this process; the angiogenic and permeable ability was significantly decreased in inhibitor cells (Figure 4G,H).

Moreover, overexpression of miR‐155‐5p in HUVECs increased the expression of VEGFA and IL‐8 and decreased VE‐cadherin expression, while knockdown of miR‐155‐5p in HUVECs significantly decreased the expression of VEGFA and IL‐8 and increased the VE‐cadherin expression (Figure 4I).

Next, the potential interaction of miR‐155‐5p with target mRNAs was analysed using TargetScan (http://www. targetscan.org/vert_72/). PIK3CA was identified as a target gene of miR‐155‐5p, and PIK3CA mRNA expression levels were decreased in OE cells and increased in inhibitor cells (Figure 4J). These data indicated that highly expressed miR‐155‐5p could enhance angiogenesis and permeability in endothelial cells.

### 3.5 MiR‐155‐5p‐enriched exosomes enhance the angiogenic and permeable activity of endothelial cells

**FIGURE 5:**
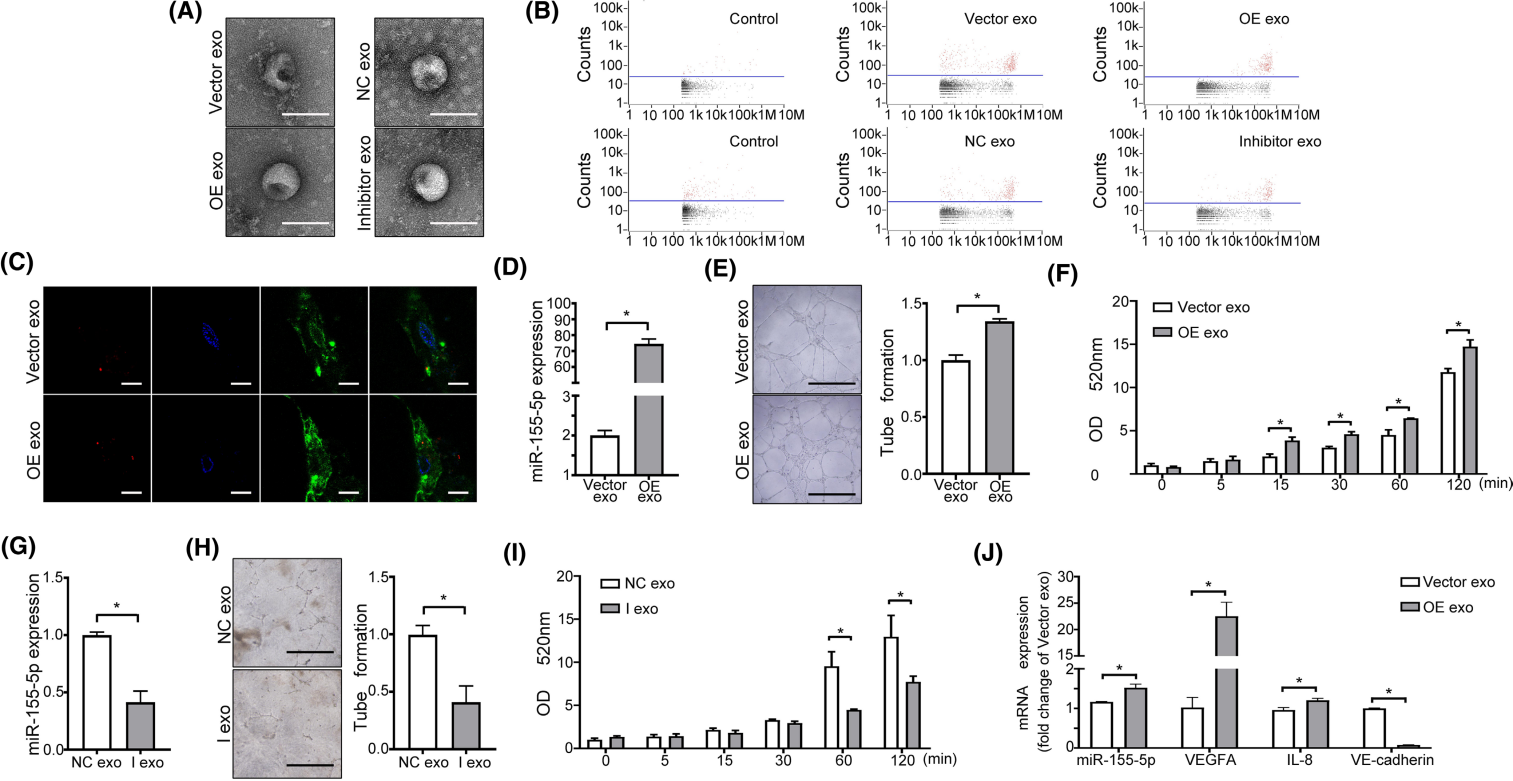
miR‐155‐5p induces angiogenesis and permeability in HAECs through exosomes. (A) Electron microscopy images of exosomes from Vector, OE, NC or Inhibitor cells (Vector exo, OE exo, NC exo and Inhibitor exo, respectively) are shown. Scale bar = 100 nm. (B) Flow cytometric analysis of CD9 and CD63 expression in Vector exo, OE exo, NC exo and Inhibitor exo. (C) Confocal images of HAECs incubated with PKH26‐labelled Vector or OE exosomes. Scale bar = 10 μm. (D) qRT‐PCR of the relative miR‐155‐5p expression level in Vector and OE exosomes. (E, F) Tube formation and permeability assays for HAECs incubated with Vector or OE exosomes. Scale bar = 500 μm. (G) The expression level of miR‐155‐5p in NC and Inhibitor exosomes was determined by qRT‐PCR. (H, I) Tube formation and permeability assays for HAECs incubated with NC or Inhibitor exosomes. Scale bar = 500 μm. (J) The qRT‐PCR analysis was used to determine miR‐155‐5p, VEGFA, IL‐8 and VE‐cadherin expression of HAECs treated with Vector exosomes and OE exosomes, respectively. All data are expressed as mean ± SD, *p < 0.05.

Electron microscopy showed that exosomes derived from Vector cells (Vector exo), OE cells (OE exo), NC cells (NC exo) and Inhibitor cells (Inhibitor exo) were quasi‐circular and smaller than 100 nm (Figure 5A). Flow cytometry further indicated that CD63 was abundant on the surface of exosomes (Figure 5B). It was also observed that HAECs internalized PKH26‐labelled exosomes (Figure 5C). Furthermore, OE exosomes significantly increased the expression of miR‐155‐5p, the number of tube‐like structures and permeability activity in HAECs (Figure 5D–F). Moreover, inhibitor exosomes decreased the expression of miR‐155‐5p, the formation of tube‐like structures and permeable ability in HAECs (Figure 5G–I). In addition, OE exosomes increased the mRNA levels of miR‐155‐5p, VEGFA and IL‐8 and decreased VE‐cadherin expression in HAECs (Figure 5J). To sum up, exosomes could change the biological functions of endothelial cells via miR‐155‐5p.

### 3.6 MiR‐155‐5p‐enriched exosomes promote atherosclerotic plaque formation

**FIGURE 6:**
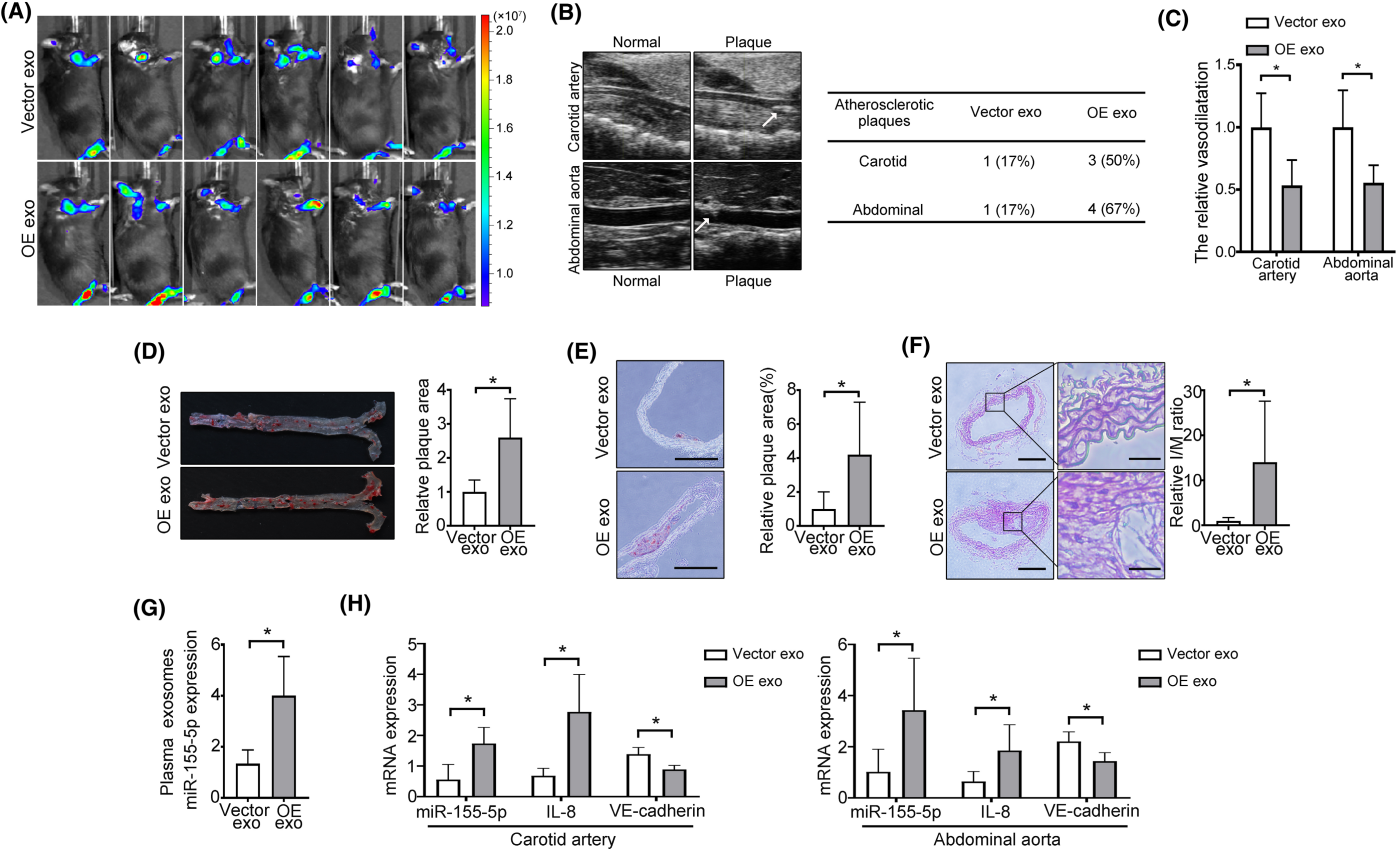
Effects of exosomal miR‐155‐5p on atherosclerotic plaque formation in vivo. (A) In vivo bioluminescence images used to study the biodistribution of PHK67‐labelled Vector exosomes or OE exosomes in mice. (B) Vascular ultrasound analysis of plaques in the carotid artery and aorta from Vector or OE exosomes group. (C) Vascular vasodilatation of the carotid artery and aorta in mice is evaluated by ultrasound and presented as changes in vasodilation and vasoconstriction diameter. (D) The atherosclerotic lesion areas in the thoracic aorta of ApoE^−/−^ mice stained with en face oil red O (left). Quantitative analysis of the plaque areas (right) is shown. (E) Representative images of oil‐red‐O‐stained aortic sections (left). Quantitative analysis of the plaque areas is shown. Scale bar = 200 μm. (F) Representative H&E‐stained images of carotid sections. Scale bar (left) = 200 μm, Scale bar (right) = 20 μm. (G) Plasma exosomes from mice in the Vector exosomes or OE exosomes group were isolated, and qRT‐PCR determined the miR‐155‐5p expression levels. (H) qRT‐PCR analysis of miR‐155‐5p, IL‐8, VE‐cadherin expression levels in the carotid artery and abdominal aorta tissues from mice. All data are expressed as mean ± SD, *p < 0.05.

Based on our findings that LPS upregulates miR‐155‐5p expression and promotes the angiogenic and permeable ability via exosomes, we sought to identify the role of exosomal miR‐155‐5p during the formation of atherosclerotic plaque in vivo. After intravenous injection of PKH67‐labelled Vector (control group) or OE exosomes, in vivo fluorescence revealed the accumulation of exosomes in the neck (Figure 6A). More atherosclerotic plaque formation was observed in the OE exosomes group compared to the control group (Figure 6B). This finding was further validated by evaluating the vasodilatation of the carotid artery and abdominal aorta (Figure 6C). Meanwhile, the thoracic aortas were covered with more plaques in mice injected with OE exosomes (Figure 6D,E). Moreover, intimal hyperplasia of the carotid artery was significantly increased in mice injected with miR‐155‐5p‐enriched OE exosomes versus the control group, and OE exosomes enhanced carotid plaque burden with more necrotic core than Vector exosomes (Figure 6F).

However, miR‐155‐5p was significantly increased in plasma exosomes stimulated with OE exosomes compared with Vector exosomes (Figure 6G). Meanwhile, OE exosomes significantly enhanced the mRNA expression of miR‐155‐5p and IL‐8 and decreased VE‐cadherin in the carotid artery and abdominal aorta tissues (Figure 6H).

### 3.7 LPS promote atherosclerotic plaque formation

**FIGURE 7:**
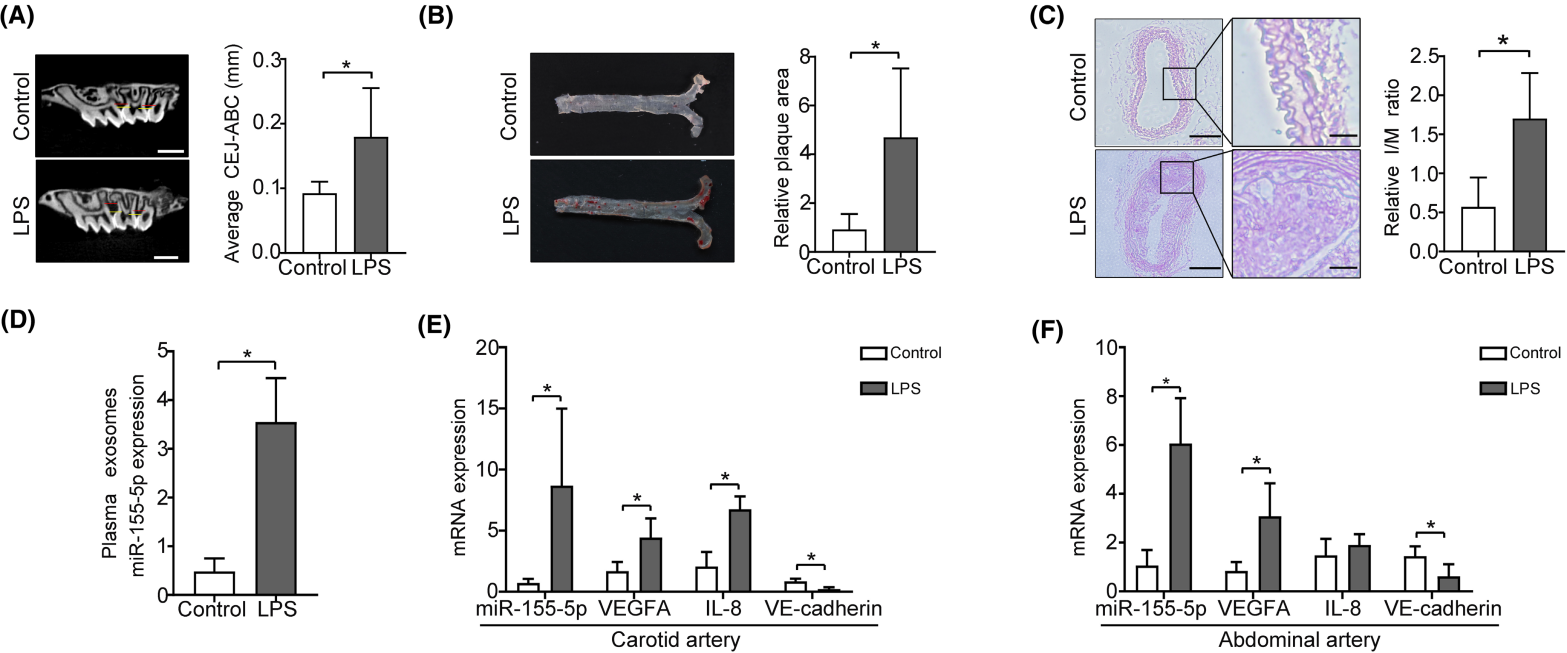
Effects of LPS on atherosclerotic plaque formation in vivo. (A) MicroCT 2D image of mouse left maxilla. Green line: The cemento‐enamel junction, Red line: The alveolar bone crest. Scale bar = 1 mm (B) The atherosclerotic lesion areas in the thoracic aorta of ApoE^−/−^ mice stained with en face oil red O (left). Quantitative analysis of the total area of plaque areas (right) is shown (*n* = 6 per group). (C) Haematoxylin–eosin (H&E) staining of tissue sections of carotid artery (*n* = 6 per group). Scale bar (left) = 200 μm, Scale bar (right) = 20 μm. (D) Plasma exosomes from mice in the Control or LPS group (*n* = 6) were isolated, and qRT‐PCR determined the miR‐155‐5p expression levels. (E, F) miR‐155‐5p, VEGFA, IL‐8, VE‐cadherin expression levels in the carotid artery and abdominal aorta tissues from mice (*n* = 6 per group) were analysed by qRT‐PCR. All data are expressed as mean ± SD, *p < 0.05.

To verify the role of LPS in occurrence of CAS, local injection of LPS into periodontal tissues was performed. The jaw specimens of mice were scanned with MicroCT, and it was found that LPS group showed significant resorption of alveolar bone toward the apical direction compared with Control group (Figure 7A). O staining of the thoracic aorta showed that there were more plaques in LPS group than Control group (Figure 7B). Moreover, intimal hyperplasia of the carotid artery was significantly increased in LPS group, and LPS could increase carotid plaque burden with necrotic core and collagens (Figure 7C).

Furthermore, miR‐155‐5p was significantly increased in plasma exosomes stimulated with LPS compared with Control (Figure 7D). LPS significantly enhanced the mRNA expression of miR‐155‐5p, VEGFA and IL‐8 and decreased VE‐cadherin in the carotid artery. Meanwhile, LPS increased the expression of miR‐155‐5p, VEGFA and decreased VE‐cadherin in abdominal aorta tissues (Figure 7E,F). The results indicated that local injection of LPS into periodontal tissues promoted CAS formation, and miR‐155‐5p played a role from periodontal microcirculation to distant CAS sites via exosomes.

## 4 DISCUSSION

MiR‐155, first found in 1997, is a small molecule for regulating the immune system.^36^ MiR‐155 has been demonstrated to be associated with the formation of CAS.^25^ Also, studies have found that miR‐155 could increase the expression of inflammatory factors such as IL‐6 and TNF‐α in endothelial cells, promoting atherosclerosis progression.^26^, ^37^ MiR‐155 can also reduce inflammation status by targeting SOCS1, while decreased miR‐155 levels can inhibite the formation of atherosclerosis.^38^, ^39^

In the present study, we found that miR‐155‐5p was overexpressed in CAS and CP plasma exosomes and was associated with the severity of CP. Further experiments revealed that LPS, enriched in periodontal pathogens, stimulated HUVECs to release exosomes with a high abundance of miR‐155‐5p. These exosomes could be absorbed by HAECs and then altered in their biological functions, including higher angiogenesis ability and permeability, which are the important pathophysiological basis of atherosclerotic plaque formation. In addition, we adopted ApoE^−/−^ mice for in vivo validation, which is currently the most widely used preclinical model of atherosclerosis.^40^ The mice that achieved miR‐155‐5p‐rich exosomes possessed a higher incidence of carotid atherosclerosis, which provided direct evidence that vascular endothelial cell‐derived exosomes promote the occurrence of carotid atherosclerosis via miR‐155‐5p.

Periodontitis is an infectious disease that occurs in the supporting tissues of teeth and is associated with several systemic diseases.^41^ In 1989, Mattila et al. first published a report on the relationship between oral infection and acute myocardial infarction,^42^ suggesting a close association between periodontitis and cardiovascular disease. At present, many clinical studies and experiments have confirmed the correlation between periodontitis and atherosclerosis, but whether there is a causal relationship between them and the exact mechanisms remains to be explored.^43^, ^44^

In vitro and in vivo studies are mainly focused on the direct effects of *P. gingivalis* or LPS on vascular endothelial cells. However, these experimental conditions only partially mimic the clinical conditions, such as transient bacteremia seen in humans. Also, these studies are more focused on persistent bacteremia and lower concentrations of LPS in peripheral blood.^11^, ^12^, ^13^, ^45^, ^46^, ^47^

In this study, we hypothesized that exosomes acted as a bridge between periodontitis and atherosclerosis. Exosomes are a kind of cystic vesicle with biological activity that have an important role in signal transmission and material exchange between cells.^20^, ^21^, ^48^ In our previous study, we proposed the term ‘exosomecrine’ to denote cell‐to‐cell signalling transduction via the exosome‐mediated transfer of molecules.^34^, ^35^ To confirm this theory, in this study, we first isolated plasma exosomes from patients and found differences between CP patients and CAS/CP patients via microRNA sequencing. Based on the conclusions from recent studies arguing that exosomes participated in the occurrence and development process of atherosclerosis,^49^, ^50^ these differences might be the molecular basis for chronic periodontitis causing carotid atherosclerosis. This exploration provides a fresh idea for the association research between CAS and CP.

According to our sequencing result, miR‐155‐5p was highly expressed in the plasma exosomes of CAS/CP patients. Consistently, we also found its' high expression in the exosomes from LPS‐treated HUVECs. A recent study also reported that miR‐155‐5p is overexpressed in mouse and human atherosclerotic lesions.^51^ Furthermore, miR‐155‐5p has already been described to target the mRNAs of central tight and adherent junction proteins, which are critical in maintaining the endothelial barrier function, and endothelial dysfunction results in atherosclerosis.^24^, ^52^ Although miR‐155‐5p has been reported to affect endothelial cells and promote atherosclerosis,^29^, ^53^ there are currently no reports on miR‐155‐5p‐mediated regulation derived from periodontitis. Similar results were obtained in this study. miR‐155‐5p‐rich exosomes derived from periodontal endothelial cells induced higher permeability of distant endothelial cells, accompanied by the decreased expression level of VE‐cadherin, ZO‐1 and Claudin‐1. In addition, miR‐155‐5p‐induced angiogenesis might be another critical process in forming atherosclerosis plaque. Interestingly, in vivo data suggested that exosomes accumulate in the neck of mice. This phenomenon might be related to the hemodynamic changes caused by the special anatomical structure of the carotid bifurcation and the increased vascular abundance during plaque formation.^54^ On the contrary, it might also suggest that exosomes have a targeting effect on the carotid artery, which is worth further exploration. Besides, oral injection of LPS increased plasma exosomal miR‐155‐5p level, suggesting miR‐155‐5p played a role from periodontal microcirculation to distant CAS sites via exosomes. Our study confirmed the role of miR‐155‐5p‐mediated exosomecrine communications between periodontitis and CAS.

However, the present study has some limitations. The occurrence of atherosclerotic is a complex process involving the participation of various cells, such as vascular endothelial cells, smooth muscle cells, monocytes and macrophages.^55^, ^56^, ^57^ The process discussed in this study is only one of the possible mechanisms through which CP leads to CAS. Another potential role of exosomal miR‐155‐5p may also be involved in CAS progression. Future studies should investigate more phenotypes and their associated mechanisms between periodontitis and CAS. Herein, we focused on the changes in HAECs after absorbing miR‐155‐5p‐rich exosomes. Moreover, the targets of miR‐155‐5p still need to be further explored. Our study suggests that exosomes derived from endothelial cells could transport highly expressed miR‐155‐5p into distant vascular endothelial cells, inducing angiogenesis ability and cell permeability increase. This process might be the mechanism through which CP causes CAS.

## 5 RECOMMENDATION AND FUTURE PERSPECTIVES

Our data suggests that miR‐155‐5p‐enriched exosomes from periodontal tissues promote carotid atherosclerosis progression, indicating that miR‐155‐5p‐enriched exosomes, which could be a therapeutic target, are a link between periodontitis and carotid atherosclerosis. Due to favourable bioavailability and biocompatibility with the characteristics of exosomes, exosomes appear to be used as drug‐delivery vehicles.^58^, ^59^ Meanwhile, drugs can be encapsulated in exosomes and transferred to target cells with minimal immune reaction and toxicity.^60^, ^61^ We hypothesized that miR‐155‐5p‐enriched exosomes are an effective vehicle for repurposing drugs and giving various vitamins such as E and D as prophylactic with tumour‐modulatory effect positively impacting atherosclerosis, endothelial function and inflammation.^62^, ^63^

## AUTHOR CONTRIBUTIONS

**Wen‐Wen Yang:** Conceptualization (equal); data curation (equal); formal analysis (equal); funding acquisition (equal); writing – original draft (equal). **Qing‐Xiang Li:** Methodology (equal); writing – original draft (equal). **Fei Wang:** Methodology (equal). **Xin‐Ran Zhang:** Software (equal). **Xian‐Li Zhang:** Resources (equal). **Meng Wang:** Visualization (equal). **Dong Xue:** Data curation (equal). **Ying Zhao:** Conceptualization (equal); funding acquisition (equal); supervision (equal); writing – review and editing (equal). **Lu Tang:** Funding acquisition (equal); supervision (equal); writing – review and editing (equal).

## FUNDING INFORMATION

This study was supported by the National Natural Science Foundation of China (grant no. 82001057).

## CONFLICT OF INTEREST STATEMENT

The authors declare that they have no competing interests.

## Supporting information


Appendix S1.

## Data Availability

All data generated or analyzed during this study are included in this published article and its Supplementary information files. All data, reagents, resources, and protocols are available from the corresponding author upon reasonable requests.
