# Supplementary material for: Exosomal miR‐155‐5p promote the occurrence of carotid atherosclerosis
Source: J Cell Mol Med. 2024 Nov 4;28(21):e70187. doi: 10.1111/jcmm.70187 (PMC11534067; doi:10.1111/jcmm.70187)
Supplement: Supplementary file 1 — Appendix S1. [file JCMM-28-e70187-s001.docx]

**Supplementary data**


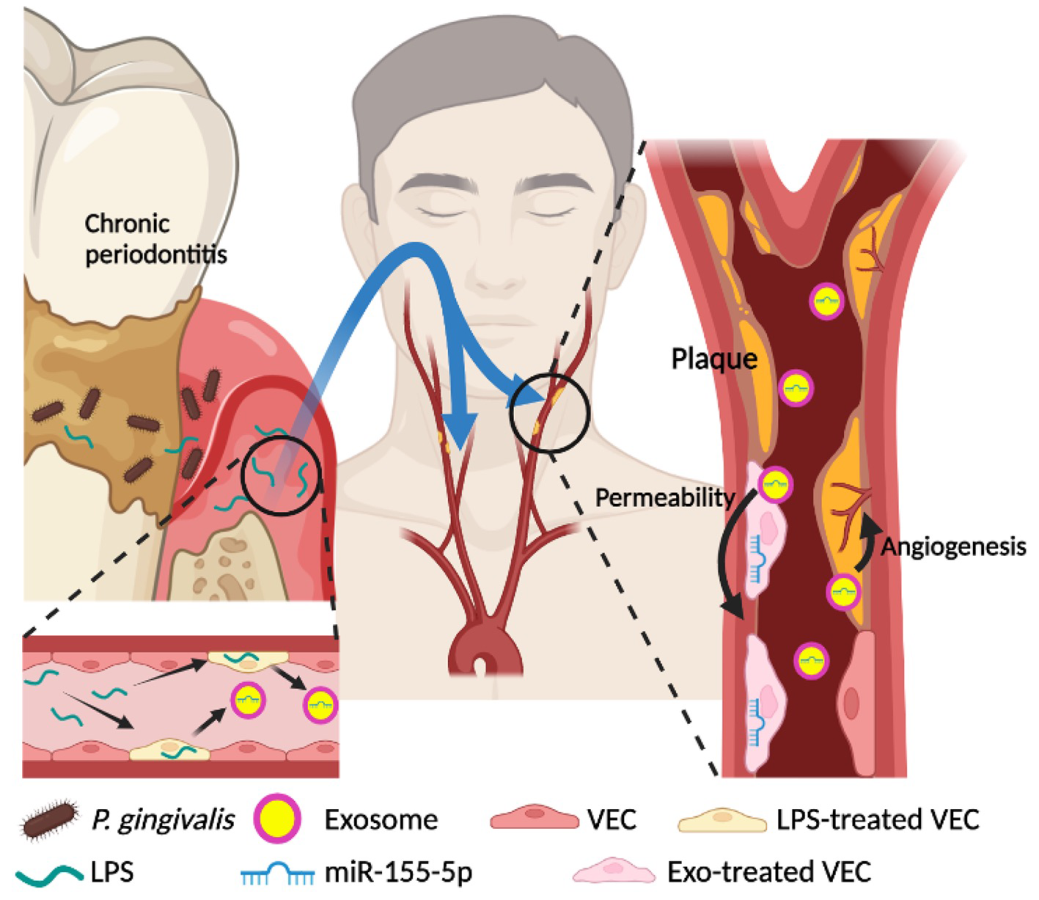


Figure S1. **Graphical Abstract**

During chronic periodontitis, LPS directly influence the periodontal endothelial cells, which results in the release of miR-155-5p-enriched exosomes to blood. The miR-155-5p-enriched exosomes could play roles in ditant carotid artery sites, increasing permeable activity and trigging the formation of angiogenesis , thus promoting the occurrence of CAS.


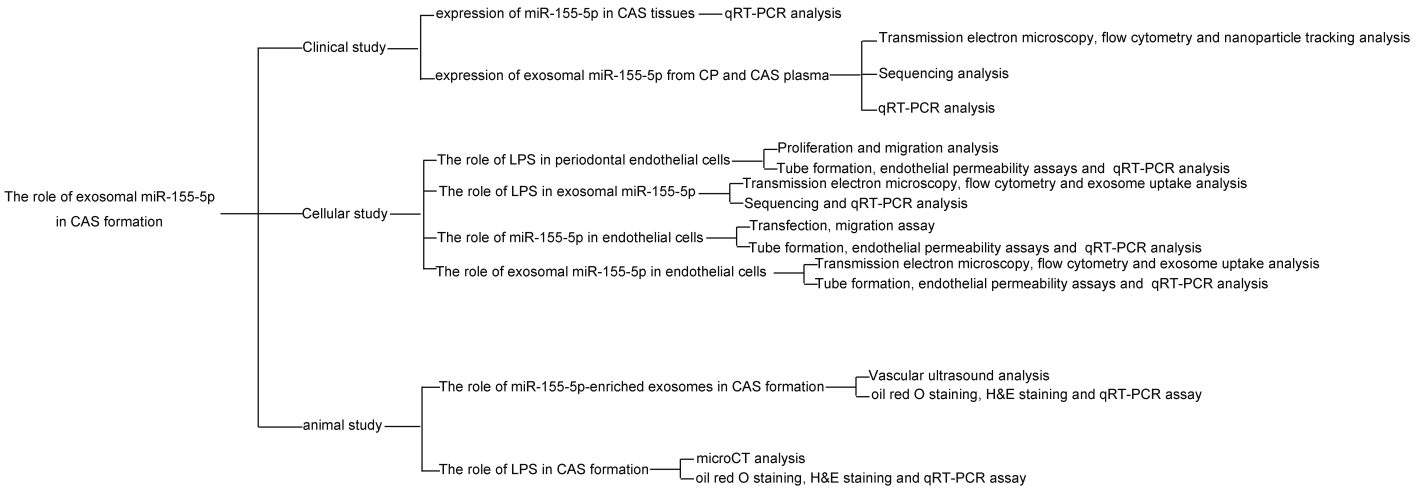


**Figure S2 The flowchart for purpose and related methods was shown.**

**
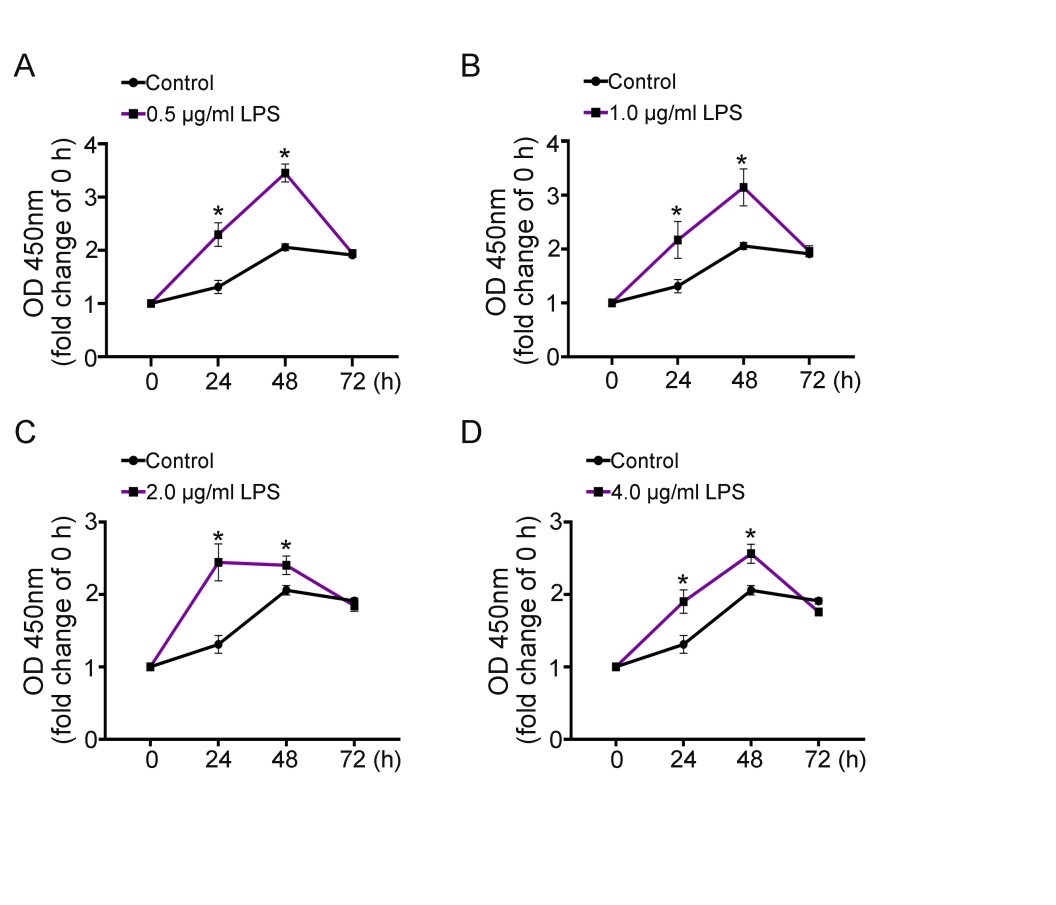
**

**Figure S3. LPS increased proliferation of HUVECs.** (A-D) CCK8 cell proliferation assays of SACC-83 cells after co-culturing with 0.5 μg/ml LPS (A), 1.0 μg/ml LPS (B), 2.0 μg/ml LPS (C) and 4.0 μg/ml LPS (D) for 24 hours, 48 hours, and 72 hours, respectively. All data are expressed as mean ± SD, **P* < 0.05.

**Tables**

**Table S1:** Primers for qRT-PCR.

| Gene | Primer sequence  (5’ to 3’) |
| --- | --- |
| qRT-PCR (human) | |
| miR-155-5p | F: CGCGTTAATGCTAATCGTGATA  R: AGTGCAGGGTCCGAGGTATT  RT:GTCGTATCCAGTGCAGGGTCCGAGGTATTCGCACTGGATACGACAACCCC |
| U6 | F: CGAATTTGCGTGTCATCCT  R: GCTTCGGCAGCACATACTAA |
| VEGFA | F: TTCTGAGTTGCCCAGGAGAC  R: GGAGCAGGAAGAGGATGAGG |
| IL-8 | F: TTTTGCCAAGGAGTGCTAAAGA  R: AACCCTCTGCACCCAGTTTTC |
| VE-cadherin | F: AAAGAATCCATTGTGCAAGTCC  R: CGTGTTATCGTGATTATCCGTG |
| GM-CSF | F: GTCTCCTGAACCTGAGTAGAGA  R: CTGGAGGTCAAACATTTCTGAG |
| MCP-1 | F: CAGCCAGATGCAATCAATGCC  R: TGGAATCCTGAACCCACTTCT |
| RANTES | F: ATCCTCATTGCTACTGCCCTC  R: GCCACTGGTGTAGAAATACTCC |
| IL-6 | F: GTGAGGAACAAGCCAGAGC  R: TACATTTGCCGAAGAGCC |
| IL-1β | F: AGCTACGAATCTCCGACCAC  R: CGTTATCCCATGTGTCGAAGAA |
| PIK3CA | F: CGGTGACTGTGTGGGACTTATTGAG  R: TGTAGTGTGTGGCTGTTGAACTGC |
| GAPDH | F: CCATGGAGAAGGCTGGG  R: CAAAGTTGTCATGGATGACC |
| qRT-PCR (mouse) | |
| miR-155-5p | F: CGCGGCCTTAATGCTAATTGTGA  R: ATCCAGTGCAGGGTCCGAGG  RT:GTCGTATCCAGTGCAGGGTCCGAGGTATTCGCACTGGATACGACACCCCT |
| U6 | F: GCTCGCTTCGGCAGCACATATAC  R: CGAATTTGCGTGTCATCCTTGCG |
| VEGFA | F: GAGCAGAAGTCCCATGAAGTGA  R: CACAGGACGGCTTGAAGATGT |
| IL-8 | F: TTTCCACCGGCAATGAAG  R: TAGAGGTCTCCCGAATTGGA |
| VE-cadherin | F: CTTCCAGCGACACTTCTACCACTTC  R: CTCTGTCACTGGTCTTGCGGATG |
| GAPDH | F: GTGTTTCCTCGTCCCGTAGA  R: AATCTCCACTTTGCCACTGC |
